# Supplementary material for: Drug repurposing for aging research using model organisms
Source: Aging Cell. 2017 Jun 16;16(5):1006–15. doi: 10.1111/acel.12626 (PMC5595691; doi:10.1111/acel.12626)
Supplement: Supplementary file 7 — Data S1 Zip‐Archive of all report cards. [file ACEL-16-1006-s007.zip › RC_DBQ.pdf]

# DBQ

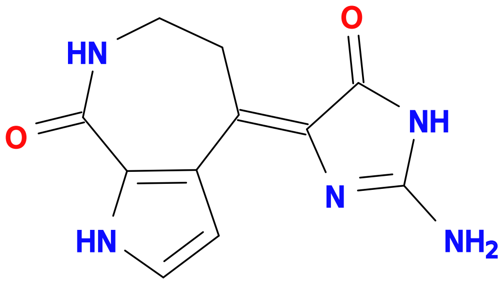


**Database identifiers**

| | |
|---|---|
| ChEMBLCompound | CHEMBL255465 |
| eMolecules | 1986433 |

## Ranking

| | Rank | Score |
|---|---|---|
| Drosophila | 368/697 | 0.405 |
| C. elegans | 293/591 | 0.195 |

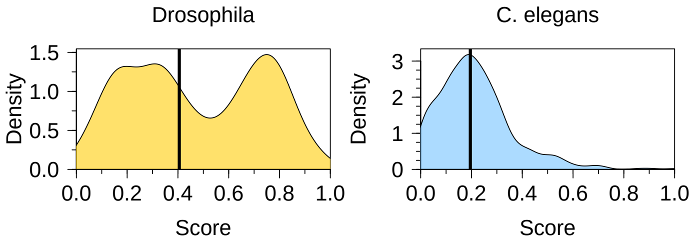


| | Ageing implication | Domain conservation | Binding site conservation | Binding affinity | Bioavailability | Lipinski | Promiscuity | Purchasability | Drug approval | **Total** |
|---|---|---|---|---|---|---|---|---|---|---|
| Drosophila | 0.563 | 0.939 | 0.868 | 0.743 | (0.9) | 0.0 | -0.001 | 0.1 | 0.0 | 0.405 |
| C. elegans | 0.563 | 0.885 | 0.952 | 0.743 | 0.275 | 0.0 | -0.001 | 0.1 | 0.0 | 0.195 |

## Names

· Debromohymenialdisine

## Roles

ChEBI entry None has no roles

## Status

| | |
|---|---|
| Approved drug (according to ChEMBL) | No |
| Number of Rule of 5 violations | 0 |
| Binding affinity to original target in log units (RF-Score prediction) | 6.06 |
| Burns *C. elegans* bioavailability prediction | -1.1 |

# Compound Target Characteristics

## Serine/threonine-protein kinase Chk1

Best gene implication in ageing for this target family came from gene O14757 annotated in UniProt release 2014_02. Annotation GO subterm of 7568 (aging) was Non-traceable Author Statement

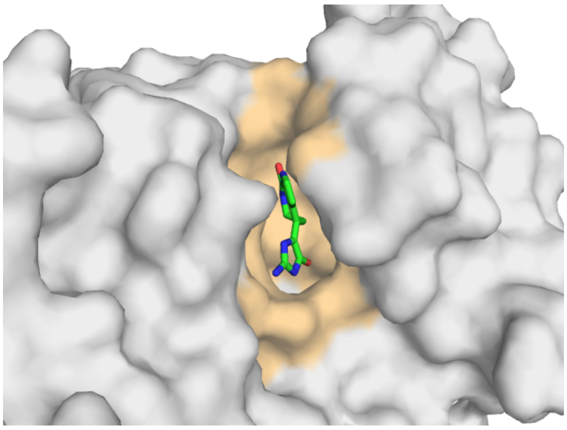

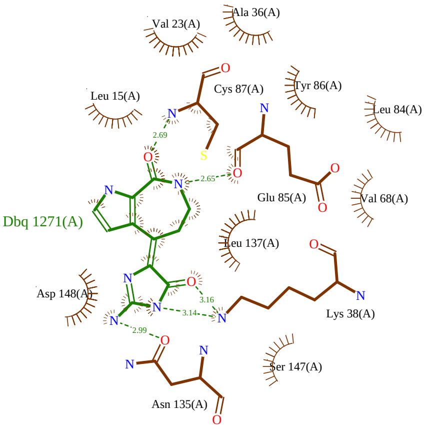


| protein | amino acids contacts (binding site) |
|---|---|
| PDB:2c3j:chainA:O14757 | L V A K V L E Y C N L S D |
| tr:E9PJI4:E9PJI4_HUMAN | L V A K V L E Y C N L S D |
| tr:E9PKQ3:E9PKQ3_HUMAN | L V A K V L E Y C N L S D |
| tr:J3KN87:J3KN87_HUMAN | L V A K V L E Y C N L S D |
| sp:O14757:CHK1_HUMAN | L V A K V L E Y C N L S D |
| sp:Q91ZN7:CHK1_RAT | L V A K V L E Y C N L S D |
| tr:G3UYB1:G3UYB1_MOUSE | L V A K V L E Y C N L S D |
| tr:G3UYC5:G3UYC5_MOUSE | L V A K V L E Y C N L S D |
| sp:O35280:CHK1_MOUSE | L V A K V L E Y C N L S D |
| sp:O61661:CHK1_DROME | L V A K L L E Y A N L S D |
| sp:Q9N3Z3:CHK1_CAEEL | L V A K I L E Y A N L S D |
| sp:P38147:CHK1_YEAST | V V A K L L E M A N L A D |

| | whole protein | | domain-based | | contact-based | |
|---|---|---|---|---|---|---|
| protein | ident | simil | ident | simil | ident | simil |
| PDB:2c3j:chainA:O14757 | 1.0 | 1.0 | 1.0 | 1.0 | 1.0 | 1.0 |
| tr:E9PJI4:E9PJI4_HUMAN | 0.36 | 0.36 | 0.63 | 0.63 | 1.0 | 1.0 |
| tr:E9PKQ3:E9PKQ3_HUMAN | 0.43 | 0.43 | 0.76 | 0.76 | 1.0 | 1.0 |
| tr:J3KN87:J3KN87_HUMAN | 0.91 | 0.91 | 1.0 | 1.0 | 1.0 | 1.0 |
| sp:O14757:CHK1_HUMAN | 1.0 | 1.0 | 1.0 | 1.0 | 1.0 | 1.0 |
| sp:Q91ZN7:CHK1_RAT | 0.94 | 0.98 | 0.98 | 1.0 | 1.0 | 1.0 |
| tr:G3UYB1:G3UYB1_MOUSE | 0.84 | 0.89 | 0.97 | 0.99 | 1.0 | 1.0 |
| tr:G3UYC5:G3UYC5_MOUSE | 0.87 | 0.91 | 0.97 | 0.99 | 1.0 | 1.0 |
| sp:O35280:CHK1_MOUSE | 0.93 | 0.98 | 0.97 | 0.99 | 1.0 | 1.0 |
| sp:O61661:CHK1_DROME | 0.44 | 0.75 | 0.56 | 0.87 | 0.85 | 0.82 |
| sp:Q9N3Z3:CHK1_CAEEL | 0.3 | 0.63 | 0.48 | 0.8 | 0.85 | 0.83 |
| sp:P38147:CHK1_YEAST | 0.23 | 0.62 | 0.35 | 0.74 | 0.62 | 0.69 |

### grp (FBgn0261278) associated phenotypes

cell cycle defective, chemical sensitive, decreased cell death, maternal effect, mitotic cell cycle defective, non-rescuable maternal effect, partially lethal - majority die, radiation resistant, radiation sensitive

(Information from FlyBase)

### grp (UniProt:O61661) annotation

**Function**: Serine/threonine-protein kinase which is required for checkpoint-mediated cell cycle arrest and activation of DNA repair in response to the presence of DNA damage or unreplicated DNA. May also negatively regulate cell cycle progression during unperturbed cell cycles. May phos-

phorylate the CDC25 phosphatase stg, which promotes its degradation. This results in increased inhibitory tyrosine phosphorylation of CDC2-cyclin complexes and consequent inhibition of cell cycle progression. (PubMed:10209095, PubMed:10469601, PubMed:10980701, PubMed:12919679, PubMed:14711410, PubMed:15723794, PubMed:15860729, PubMed:16079276, PubMed:7925016, PubMed:9197245, PubMed:9214509).
**Subcellular location**: Nucleus ECO:0000269—PubMed:10980701, PubMed:16079276).
**Developmental stage**: Expressed both maternally and zygotically. Maternally supplied mRNA is degraded during progression from nuclear stage 12 to nuclear stage 13. Zygotic expression is seen at reduced levels later in embryogenesis and during larval development. Higher expression is seen in pupae, coincident with ovarian differentiation. May be activated during the syncytial blastoderm divisions which precede cellularization, the Drosophila equivalent of the midblastula transition (MBT). Developmentally regulated activation of the DNA replication checkpoint may occur as the nucleo-cytoplasmic ratio increases and maternal replication factors are depleted. Elongation of the embryonic cell cycle may allow time for the transcription of genes that initiate the switch from maternal to zygotic control of embryogenesis. (PubMed:9197245, PubMed:9214509).
**Ptm**: Phosphorylated in a MEI-41/ATR dependent manner in response to DNA damage or the presence of unreplicated DNA. (PubMed:15860729).

(Information from UniProt)

### chk-1 (WBGene00000498) associated phenotypes

lethal, locomotion variant, mid larval lethal, sterile

(Information from WormBase)

### chk-1 (UniProt:Q9N3Z3) annotation

**Function**: Serine/threonine-protein kinase which is required for checkpoint-mediated cell cycle arrest and activation of DNA repair in response to the presence of DNA damage or unreplicated DNA. May also negatively regulate cell cycle progression during unperturbed cell cycles. Required for checkpoint mediated cell cycle arrest in response to DNA damage in germline cells. Essential for embryogenesis. (PubMed:15326393).
**Subcellular location**: Cytoplasm Nucleus
**Tissue specificity**: Expressed in the germline. (PubMed:15326393).
**Developmental stage**: Highly expressed in the embryo. (PubMed:15326393).

(Information from UniProt)

## Serine/threonine-protein kinase Chk2

Best gene implication in ageing for this target family came from gene O96017 annotated in UniProt release 2014_02. Annotation GO subterm of 7568 (aging) was Non-traceable Author Statement

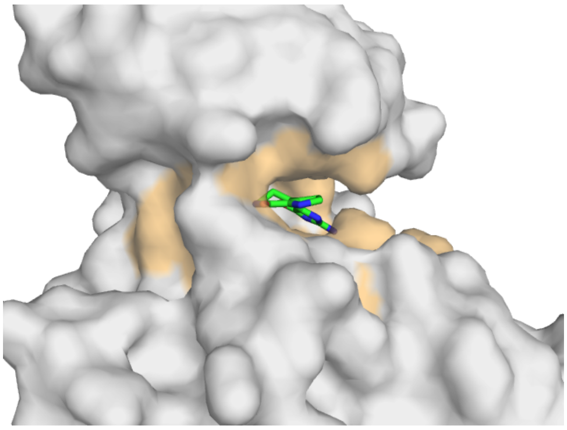

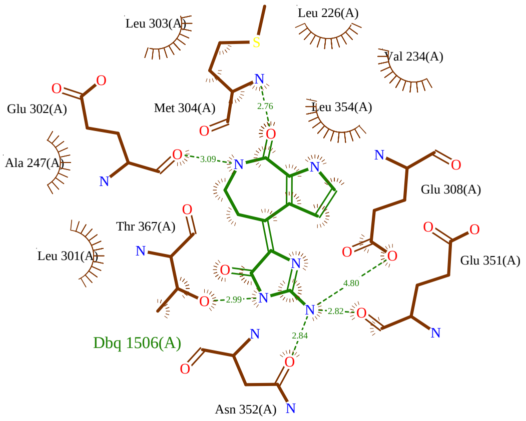

| protein | amino acids contacts (binding site) |
|---|---|
| PDB:2cn8:chainA:O96017 | L V A K L E L M E E N L T |
| sp:O96017:CHK2_HUMAN | L V A K L E L M E E N L T |
| tr:Q9R019:Q9R019_RAT | L V A K L E L M E E N L T |
| sp:Q9Z265:CHK2_MOUSE | L V A K L E L M E E N L T |
| tr:Q543W6:Q543W6_MOUSE | L V A K L E L M E E N L T |
| tr:A4V0X0:A4V0X0_DROME | L V A K L E F M D D N L S |
| sp:O61267:LOK_DROME | L V A K L E F M D D N L S |
| tr:A4F324:A4F324_CAEEL | L V A K I E Y V E E N L T |
| sp:Q9U1Y5:CHK2_CAEEL | L V A K I E Y V E E N L T |
| sp:P22216:RAD53_YEAST | V V A K M E F V D D N L T |

| | whole protein | | domain-based | | contact-based | |
|---|---|---|---|---|---|---|
| protein | ident | simil | ident | simil | ident | simil |
| PDB:2cn8:chainA:O96017 | 1.0 | 1.0 | 1.0 | 1.0 | 1.0 | 1.0 |
| sp:O96017:CHK2_HUMAN | 1.0 | 1.0 | 1.0 | 1.0 | 1.0 | 1.0 |
| tr:Q9R019:Q9R019_RAT | 0.82 | 0.92 | 0.92 | 0.98 | 1.0 | 1.0 |
| sp:Q9Z265:CHK2_MOUSE | 0.82 | 0.92 | 0.92 | 0.98 | 1.0 | 1.0 |
| tr:Q543W6:Q543W6_MOUSE | 0.82 | 0.92 | 0.92 | 0.98 | 1.0 | 1.0 |
| tr:A4V0X0:A4V0X0_DROME | 0.31 | 0.65 | 0.43 | 0.81 | 0.69 | 0.87 |
| sp:O61267:LOK_DROME | 0.3 | 0.63 | 0.43 | 0.81 | 0.69 | 0.87 |
| tr:A4F324:A4F324_CAEEL | 0.27 | 0.62 | 0.36 | 0.75 | 0.77 | 0.95 |
| sp:Q9U1Y5:CHK2_CAEEL | 0.27 | 0.62 | 0.36 | 0.75 | 0.77 | 0.95 |
| sp:P22216:RAD53_YEAST | 0.16 | 0.39 | 0.38 | 0.73 | 0.54 | 0.86 |

### lok (FBgn0019686) associated phenotypes

cell death defective, chemical resistant, conditional, decreased cell death, increased cell death, long lived, maternal effect, radiation sensitive

(Information from FlyBase)

### lok (UniProt:O61267) annotation

**Function**: May have a role in germline establishment. (PubMed:9507063).
**Subcellular location**: Nucleus speckle(PubMed:9507063). Note=Speckled subnuclear compartment.
**Tissue specificity**: In stage 3 embryos, both isoforms are expressed in both somatic and pole cell nuclei. Expression in pole cell nuclei is sustained until stage 9 and weakly expressed after pole cell invagination into the abdominal cavity. (PubMed:9507063).
**Developmental stage**: Expressed both maternally and zygotically in adult females. Levels of the long isoform remain fairly constant from ovaries to embryos, the levels of short isoform decrease dramatically. (PubMed:9507063).

(Information from UniProt)

### chk-2 (UniProt:Q9U1Y5) annotation

**Function**: Serine/threonine-protein kinase which is required for checkpoint-mediated cell cycle arrest, activation of DNA repair and apoptosis in response to the presence of DNA double-strand breaks. May also negatively regulate cell cycle progression during unperturbed cell cycles. Phosphorylates and inhibits cdc25 phosphatase, preventing entry into mitosis. Required for nuclear reorganization and homologous chromosome pairing during meiotic prophase. (PubMed:11086161, PubMed:11158318).
**Cofactor**: Mg(2+)
**Subcellular location**: Nucleus (PubMed:11086161, PubMed:11158318).
**Tissue specificity**: Highly expressed in germline tissue. (PubMed:11086161).
**Developmental stage**: Low expression in embryos and L2 larvae, high expression in hermaphrodite adults. (PubMed:11086161).

(Information from UniProt)
